# Case report: 10-year follow-up of a patient with neuronal intranuclear inclusion disease and a literature review

**DOI:** 10.3389/fnins.2024.1530160

**Published:** 2025-01-15

**Authors:** Kenji Yoshida, Tomotsugu Kaga, Sachiko Hosoyama, Jun-ichi Niwa, Jun Sone, Naoki Mabuchi

**Affiliations:** ^1^Department of Neurology, Nagoya Ekisaikai Hospital, Nagoya, Japan; ^2^Department of Neurology, Aichi Medical University, Nagakute, Japan; ^3^Department of Neuropathology, Institute for Medical Science of Aging, Aichi Medical University, Nagakute, Japan

**Keywords:** neuronal intranuclear inclusion disease, magnetic resonance imaging, dementia, *NOTCH2NLC*, neurodegenerative disease

## Abstract

Neuronal intranuclear inclusion disease (NIID) is a rare, progressive neurodegenerative disease with variable clinical manifestations. High signals on diffusion-weighted imaging (DWI) along the corticomedullary junction (CMJ) are a specific feature of NIID. Only a few reports have observed patients for a long period and demonstrated a relationship between magnetic resonance imaging (MRI) features and clinical manifestations. Herein, we present a case of a patient with NIID who underwent a 10-year brain MRI follow-up study and a literature review. A 78-year-old woman presented with severe cognitive dysfunction and disturbances of consciousness. Her brain MRI DWI signal intensity gradually increased over 10 years, and her cognitive function progressively declined. The DWI signal changes were related to the clinical manifestations in this case. In the literature review, we analyzed patients with NIID by classifying them into subgroups and found that high signals on fluid-attenuated inversion recovery (FLAIR) and DWI were related to dementia. Although high DWI signals along the CMJ are specific to NIID, many patients also show high signals on FLAIR in the deep subcortical white matter. In our literature review, dementia could have some correlation to MRI signals. In our case with longitudinal follow up, the DWI high intensity signal expansion could have correlation to cognitive decline. We found dementia and the dementia progression may have some relation to expansion of DWI with intensity signals from the CMJ to the deep subcortical white matter. Our report highlights that DWI signal changes are strongly correlated with the clinical manifestations of NIID.

## 1 Introduction

Neuronal intranuclear inclusion disease (NIID) is a rare, chronic, and progressive neurodegenerative disease with complex and diverse clinical manifestations (Lindenberg et al., 1968; Schuffler et al., 1978; Michaud and Gilbert, 1981). NIID shows a pathological eosinophilic intranuclear inclusion complex in the central and peripheral nervous systems and multiple visceral organs. High signal intensity in the corticomedullary junction (CMJ) on diffusion-weighted imaging (DWI) is a key characteristic feature of NIID. Despite these features, a definite diagnosis of NIID is difficult because of the wide range of associated neurological and systemic disorders. Dementia, muscle weakness, parkinsonism, and autonomic dysfunction are common in NIID (Takahashi-Fujigasaki, 2003; Sone et al., 2016), and some patients with NIID present episodic symptoms such as encephalitis and stroke (Xie et al., 2022). Because NIID cases show variable clinical manifestations, they can be easily misdiagnosed as other diseases. The expansion of the GGC repeat sequence in the *NOTCH2NLC* gene causes NIID (Deng et al., 2019; Ishiura et al., 2019; Sone et al., 2019; Tian et al., 2019). These findings, combined with brain magnetic resonance imaging (MRI) studies, contribute to the rigid diagnosis of NIID and reveal that, in several cases, patients with NIID did not show high-intensity DWI signals on the CMJ. Although several clinical studies have investigated the relationship between the number of GGC repeat sequences and clinical symptoms, age of onset, and MRI features (Tai et al., 2023), the MRI features affected by variable clinical manifestations have not been clearly elucidated. Herein, we report an adult-onset case of NIID, followed by MRI for 10 years. We also reviewed all published NIID cases which were positive for GGC repeat sequence expansion with MRI studies.

## 2 Case description

### 2.1 Study participant

We present a case of NIID diagnosed using *NOTCH2NLC* gene testing and skin biopsy, with MRI DWI showing high linear intensity signals in the CMJ, followed by MRI over 10 years. This study included a case of NIID in Japan. The patient was recruited from our hospital. The patient exhibited typical linear signals in the CMJ on DWI and underwent skin biopsy and *NOTCH2NLC* gene testing to confirm the diagnosis. The Japanese versions of the Montreal Cognitive Assessment (MoCA-J) and the Mini-Mental State Examination (MMSE) were used to screen for cognitive impairment. Subsequently, the patient underwent a lumbar puncture, nerve conduction study (NCS) test, and electroencephalography (EEG).

### 2.2 Case presentation

**Figure 1:**
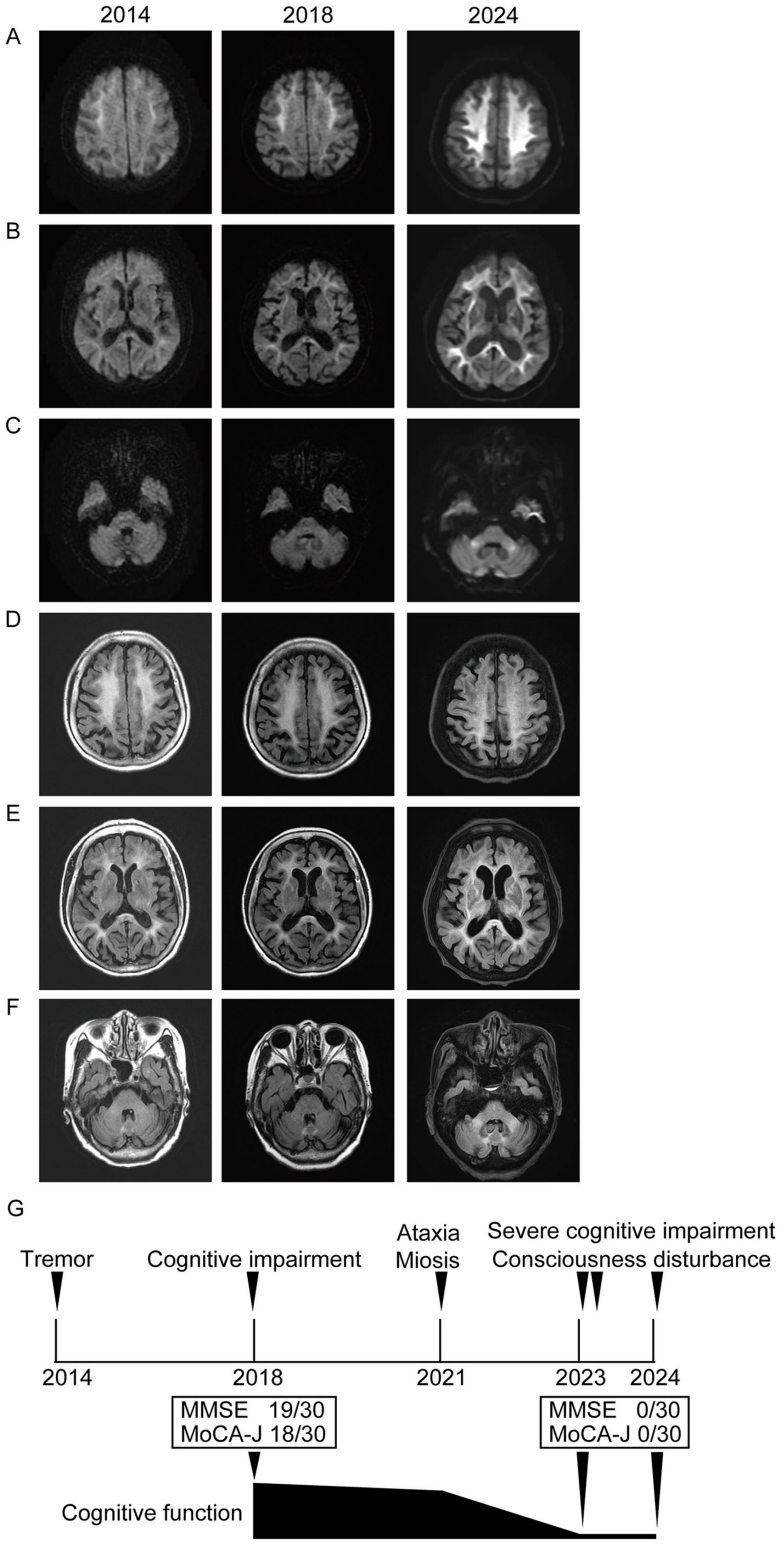
**(A–C)** Brain MRI images on DWI are shown. Each image was taken in 2014, 2018, and 2024. **(A,B)** DWI high-intensity signals were observed along CMJ, and they expanded to deep white matter. **(C)** DWI high-intensity signals were observed in cerebellar peduncles in 2024. **(D–F)** Brain MRI images on FLAIR are shown. Each image was taken in 2014, 2018, and 2024. **(D,E)** FLAIR high-intensity signals were observed in deep white matter in 2014, 2018, and 2024. **(F)** FLAIR high-intensity signals were observed in paravermis from 2014 to 2024. It was observed in bilateral cerebellar peduncles in 2024. **(G)** Schematic diagram of the timeline and clinical manifestation of our case. MRI, magnetic resonance imaging; DWI, diffusion-weighted imaging; FLAIR, fluid-attenuated inversion recovery; MoCA-J, Montreal Cognitive Assessment; MMSE, Mini-Mental State Examination.

**Figure 2:**
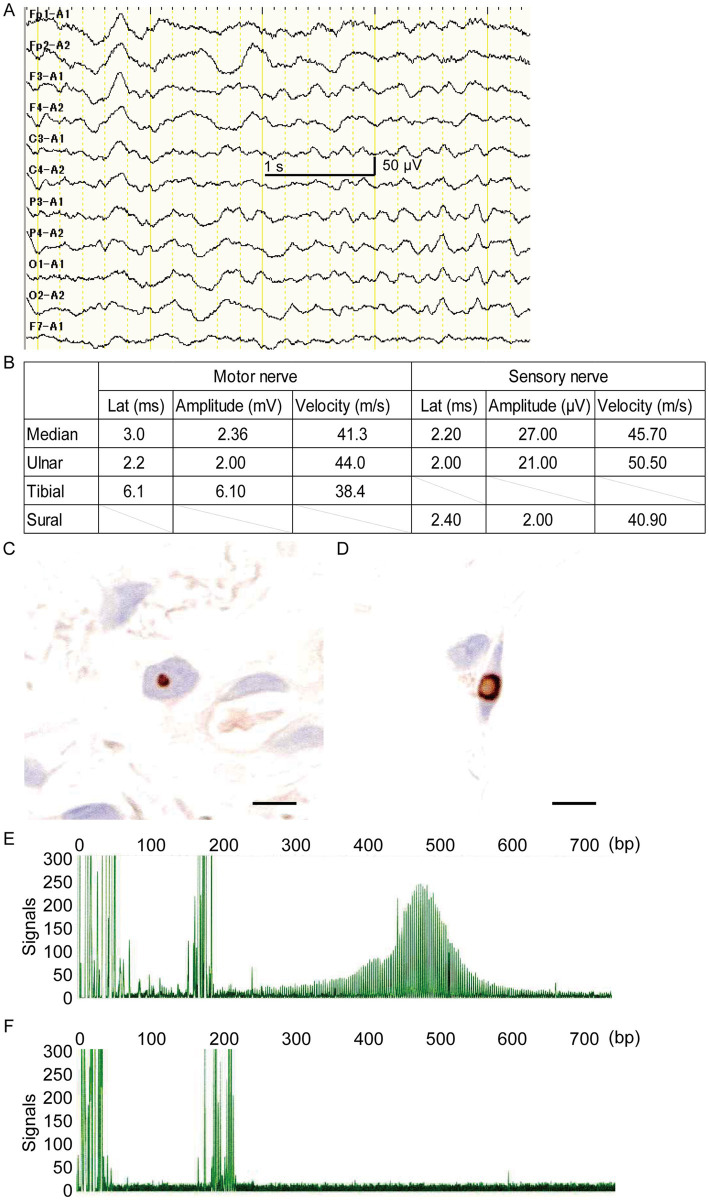
**(A)** Electroencephalogram (EEG) of this case is shown. The EEG frequency indicated about 6–7 Hz, theta band oscillation (scale bar represents 1.0 s and 50 μV). **(B)** Results of the nerve conduction velocity study are shown. Motor nerve conduction velocity in the median, ulnar, and tibial nerves, as well as sensory nerve conduction velocity in the median nerve, ulnar, and sural nerves, are shown. The amplitude was slightly decreased, and the latency was slightly increased, suggesting axonal neuropathies existed. **(C)** Skin biopsy findings with anti-p62 antibody immunostaining of fibroblasts (scale bar represents 10 μm). **(D)** Skin biopsy findings with anti-p62 antibody immunostaining of dermal adipocyte (scale bar represents 10 μm). **(E)** Expansion of the GGC repeat on the *NOTCH2NLC* gene was detected by florescence amplicon analysis. **(F)** Control gene sequences of control by florescence amplicon analysis.

A 78-year-old right-handed woman was admitted to our hospital in 2023 with altered consciousness and cognitive decline. In 2014, she experienced postural tremors in both upper limbs. High-intensity signals on DWI were observed in the CMJ (Figure 1A), and high-intensity signals on FLAIR were observed in the white matter (Figure 1D). The patient was diagnosed with essential tremor. In 2018, her cognitive function began declining, and the MMSE and MoCA-J scores were 19/30 and 18/30, respectively. Since DWI high-intensity signals were observed along the CMJ (Figures 1A,B), the diagnosis of NIID was considered; however, the patient and her family declined a skin biopsy at that time to diagnose NIID. In 2021, she developed autonomic symptoms, such as miosis. She exhibited ataxia and progressive cognitive decline and was subsequently admitted to our hospital. The patient exhibited limb tremors, and the finger-nose and heel–knee tests were poorly coordinated bilaterally. She could barely walk and was unable to perform tandem gait owing to her truncal ataxia. High-intensity signals on DWI and FLAIR were observed in the paravermal region (Figures 1C,F). In 2023, she experienced consciousness disturbances similar to an encephalitic disorder, and she was admitted to our hospital. She also had fever of unknown origin. The consciousness disturbance was transient and resolved after several days of follow-up without medical treatment. Between 2023 and 2024, she experienced transient encephalitis-like symptoms and consciousness disturbances several times, resulting in multiple hospital admissions. In 2024, the high-intensity signals on DWI expanded to the deep subcortical white matter. High-intensity signals on DWI and FLAIR were observed in both cerebellar peduncles, suggesting MRI findings were consistent with the clinical manifestations (Figures 1C,F). A routine complete blood count and serum biochemistry revealed strong inflammatory responses. Cerebrospinal fluid (CSF) pressure and routine CSF analysis showed no significant abnormalities. Electrocardiography showed normal sinus rhythm. EEG revealed a diffuse slow background (Figure 2A). NCS revealed slightly slow conduction velocity and low amplitude in both the motor and sensory nerves, suggesting mild nerve axon and myelin damage (Figure 2B). No significant abnormalities were observed on chest or abdominal computed tomography. High-intensity signals on DWI were observed along the CMJ of both hemispheres and in the deep subcortical white matter (Figures 1A,B). FLAIR images also showed diffuse high-intensity signals in the deep white matter (Figures 1D,E). A longitudinal brain MRI study revealed that high-intensity signals on DWI expanded from the CMJ to the deep white matter, whereas high-intensity FLAIR signals had already been observed in the deep subcortical white matter at first admission (Figures 1A,D). NIID was strongly suspected based on the clinical manifestations and MRI findings. A skin biopsy was performed, and intranuclear inclusion bodies were observed (Figures 2C,D). We investigated the segregation of GGC repeat expansions in the genome using repeated primed polymerase chain reaction, which demonstrated the repeat expansion in a sawtooth pattern in the patient. The total number of GGC repeats was 94 (Figures 2E,F), which was higher than normal. Based on the clinical manifestations, MRI studies, electrophysiological studies, and genetic studies, we diagnosed the patient with NIID.

In 2014, high-intensity signals on DWI were observed only in the CMJ; however, diffuse high-intensity FLAIR signals were shown in the deep subcortical white matter. In this case, the cognitive function declined as the DWI signals spread from the CMJ to the deep white matter. This suggests that MRI signal changes correlate with clinical manifestations, especially the progression of cognitive impairment (Figures 1A,G).

## 3 Literature review

### 3.1 Patients

We also reviewed all published NIID cases positive for *NOTCH2NLC* GGC repeat expansion and skin biopsy results in the PubMed database by searching the terms “neuronal intranuclear inclusion disease.” Case reports and reviews were obtained and examined. The inclusion criteria were as follows: (1) skin biopsy indicating intranuclear inclusions in the nuclei of fibroblasts, fat cells, and ductal epithelial cells of sweat glands; (2) *NOTCH2NLC* gene testing showing abnormal GGC repeat sequence expansion; (3) clinical symptoms; and (4) brain MRI studies with at least DWI and fluid-attenuated inversion recovery (FLAIR). Finally, 112 patients met the inclusion criteria (Okubo et al., 2019; Guo et al., 2020; Ishihara et al., 2020; Li et al., 2020; Liang et al., 2020; Deng et al., 2021; Huang et al., 2021; Pang et al., 2021; Cao et al., 2022; Liao et al., 2022; Wang et al., 2022; Liu et al., 2023; Wang and Qiu, 2023; Xu et al., 2023; Ishizawa et al., 2024; Lee et al., 2024; Miyaue et al., 2024; Yu et al., 2024). Patients were divided into the following subgroups: dementia, movement disorders, autonomic failure, and episodic symptoms. The patients were divided into four populations using the hierarchical clustering method, supported by clinical symptoms. Case reports and reviews were analyzed using MATLAB software packages (MathWorks, Natick, MA, United States).

### 3.2 Statistical analysis

GraphPad Prism 10 software (GraphPad Software Inc., La Jolla, CA, United States) was used for statistical analysis. All data are presented as mean ± standard error of deviation. Kruskal–Wallis test followed by Dunn’s tests, chi-square test and simple linear regression were used to assess statistical significance. Statistical significance was set at *p* < 0.05. A hierarchical clustering analysis was performed to identify clusters among 112 cases using 22 clinical symptoms listed in Table 1 as variables by MATLAB software packages (Math Works, Natick, MA, United States). This method starts with each participant as its own cluster, combining the most similar participants based on closeness. Several solutions between 3 and 10 clusters were considered, with a final 4-cluster solution selected.

**Table 1:** Clinical manifestations of NIID.

	Total		Dementia		Movement Disorder		Autonomic failure		Episodic symptom	
Sex ratio (ratio of women)	56.25		57.14		73.91		44.83		53.85	
Age of onset (years; 14 to 86)	58.03 ± 13.08 (14 to 86)		56.71 ± 15.67 (18 to 86)		51.78 ± 15.50 (14 to 72)		60.10 ± 10.42 (27 to 76)		60.87 ± 10.72 (31 to 80)	
Total (*n* = 112)	*n* = 112	(%)	*n* = 21	(%)	*n* = 23	(%)	*n* = 29	(%)	*n* = 39	(%)
Cognitive impairment	68	60.71	21	100.00	2	8.70	24	82.76	21	53.85
Headache	26	23.21	5	23.81	3	13.04	8	27.59	10	25.64
Dizziness	10	8.93	1	4.76	2	8.70	4	13.79	3	7.69
Vision disorder	12	10.71	2	9.52	5	21.74	1	3.45	4	10.26
Ataxia	22	19.64	0	0.00	7	30.43	5	17.24	10	25.64
Movement disorder	45	40.18	13	61.90	16	69.57	13	44.83	3	7.69
Tremor	43	38.39	12	57.14	16	69.57	12	41.38	3	7.69
Rigidity	8	7.14	4	19.05	3	13.04	1	3.45	0	0.00
Bradykinesia	3	2.68	1	4.76	0	0.00	2	6.90	0	0.00
Autonomic	45	40.18	2	9.52	2	8.70	29	100.00	12	30.77
Urinary disturbance	29	25.89	0	0.00	2	8.70	17	58.62	10	25.64
Constipation	6	5.36	0	0.00	0	0.00	4	13.79	2	5.13
Syncope	3	2.68	0	0.00	0	0.00	2	6.90	1	2.56
Miosis	16	14.29	1	4.76	0	0.00	12	41.38	3	7.69
Weakness	63	56.25	11	52.38	11	47.83	23	79.31	18	46.15
Peripheral neuropathy	30	26.79	6	28.57	5	21.74	14	48.28	5	12.82
Muscle weakness	37	33.04	6	28.57	8	34.78	12	41.38	11	28.21
Sensory disturbance	19	16.96	1	4.76	5	21.74	8	27.59	5	12.82
Episodic symptom	54	48.21	3	14.29	2	8.70	10	34.48	39	100.00
Encephalitic episode	39	34.82	2	9.52	1	4.35	5	17.24	31	79.49
Disturbance of consciousness	22	19.64	1	4.76	1	4.35	6	20.69	14	35.90
Stroke-like episode	8	7.14	1	4.76	0	0.00	0	0.00	7	17.95
	***n***	**Score**	***n***	**Score**	***n***	**Score**	***n***	**Score**	***n***	**Score**
MMSE (2 to 29)	59	20.75 ± 6.870 (2 to 29)	14	20.50 ± 6.700 (12 to 28)	10	26.90 ± 1.969 (24 to 29)	18	17.83 ± 6.947 (2 to 28)	17	20.41 ± 6.911 (7 to 28)
MoCA (9 to 27)	17	19.06 ± 5.190 (9 to 27)	7	15.43 ± 5.412 (9 to 21)	5	22.80 ± 3.421 (20 to 27)	3	21.67 ± 5.515 (19 to 24)	2	18.50 ± 3.536 (16 to 21)
FAB (2 to 15)	16	9.63 ± 4.303 (2 to 15)	6	8.83 ± 3.647 (5 to 14)	0	N.A.	6	8.67 ± 4.967 (2 to 15)	4	12.25 ± 4.272 (6 to 15)
	***n* = 112**	**(%)**	***n* = 21**	**(%)**	***n* = 23**	**(%)**	***n* = 29**	**(%)**	***n* = 39**	**(%)**
DWI	92	82.14	19	90.48	19	82.61	25	86.21	29	74.36
FLAIR	79	70.54	17	80.95	14	60.87	27	93.10	21	53.85
Ventricular distention	27	24.11	6	28.57	3	13.04	13	44.83	5	12.82

DWI, diffusion-weighted imaging; FAB, frontal assessment battery; FLAIR, fluid-attenuated inversion recovery; MoCA-J, Montreal Cognitive Assessment; MMSE, Mini-Mental State Examination. The mean ± standard deviation (SD) and range for continuous variables are presented.

## 4 Result

**Figure 3:**
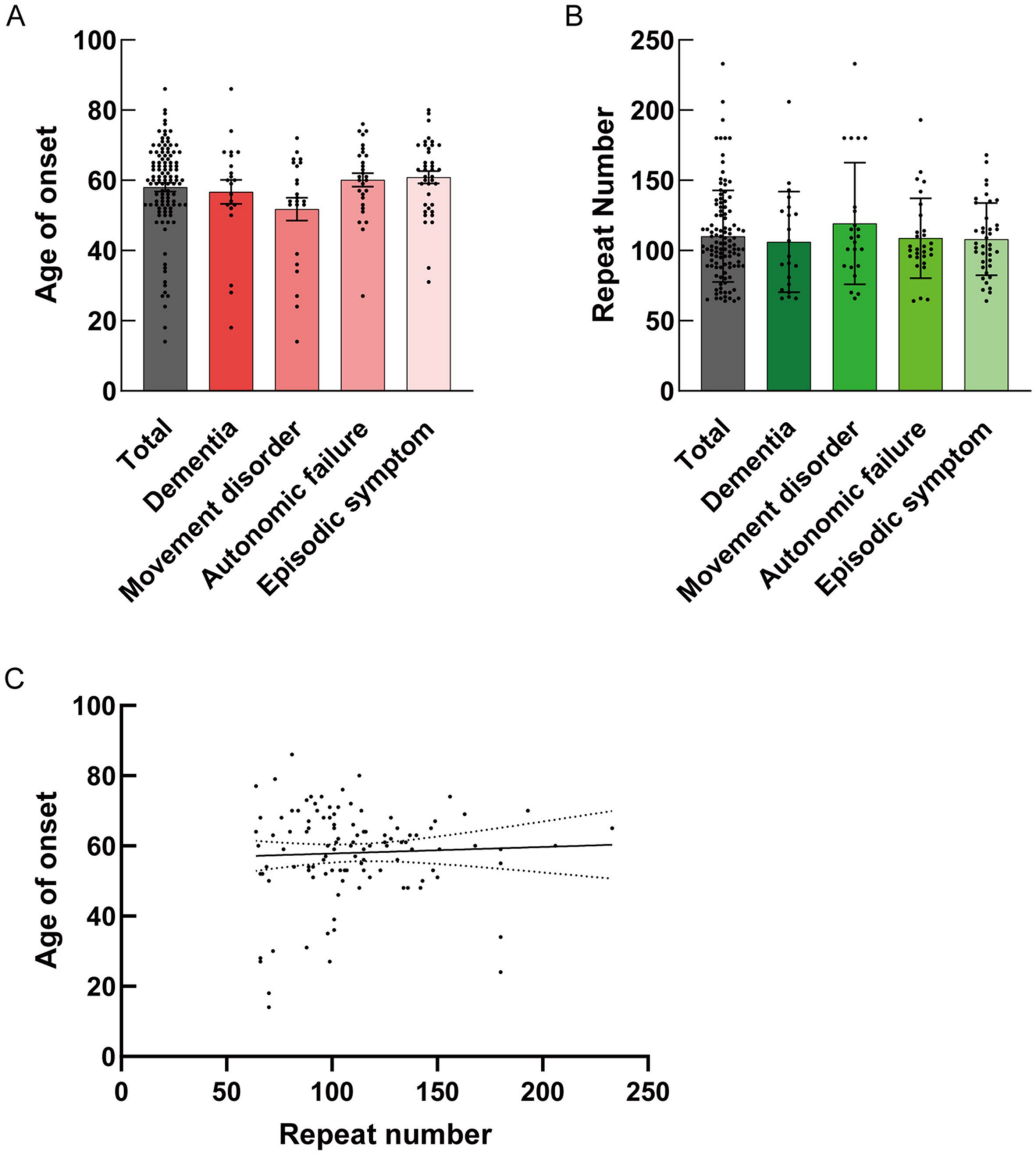
**(A)** Mean size ± standard deviation (SD) of onset age for each clinical manifestation is shown. No statistical differences were detected in the mean size ± SD of onset age along with clinical manifestation by the Kruskal–Wallis test (Total: *N* = 112, 58.03 ± 13.08; Dementia: *N* = 21, 56.71 ± 15.67; Movement disorder: *N* = 23, 51.78 ± 15.50; Autonomic failure: *N* = 29, 60.10 ± 10.42; Episodic symptom: *N* = 39, 60.87 ± 10.72). **(B)** Mean size ± SD of the GGC repeat number for each clinical manifestation is shown. No statistical differences were detected in the mean size ± SD of the GGC repeat number along with clinical manifestation by Kruskal–Wallis test (Total: *N* = 112, 110.2 ± 32.52; Dementia: *N* = 21, 106.2 ± 35.83; Movement disorder: *N* = 23, 119.2 ± 43.33; Autonomic failure: *N* = 29, 108.8 ± 28.51; Episodic symptom: *N* = 39, 108.1 ± 25.77). **(C)** Statistical analysis of the correlation between onset age and repeat number by simple linear regression (*Y* = 0.01862*X* + 55.97, *R* squared = 0.002146, Sy.x = 13.12, *p* = 0.6277).

The clinical characteristics of the 112 patients with NIID, including the present case, are summarized in Table 1. Patients with NIID usually presented in adulthood, with a mean age of 58.03 years. The most common symptoms among the 112 patients were cognitive impairment (60.71%), with an MMSE score of 20.75, MoCA score of 19.06, and Frontal Assessment Battery score of 9.63, followed by autonomic dysfunction (40.18%), tremors (38.39%), encephalitic episodes (34.82%), muscle weakness (33.04%), peripheral neuropathy (26.79%), headache (23.21%), and ataxia (19.64%). Among the 112 patients, 92 (82.14%) showed high-intensity signals in the CMJ on DWI, and 79 (70.54%) showed leukoencephalopathy on FLAIR. Only 27 patients (24.11%) showed ventricular distension. Based on clinical manifestations, we categorized the 112 cases into dementia (*n* = 21), movement disorder (*n* = 23), autonomic failure (*n* = 29), and episodic symptoms (*n* = 39) using the hierarchical clustering method. In the dementia group, 19 of 21 patients (90.48%) showed high-intensity signals in the CMJ on DWI; however, in other groups, the DWI study showed that only 82.61% (movement disorder), 86.21% (autonomic failure), and 74.36% (episodic symptoms) of patients had high-intensity signals. No significant differences were identified among the four subgroups in DWI U-fiber high-intensity signals using the chi-squared test (*p* > 0.05) (Table 1). In the brain FLAIR study, 17 of 21 patients (80.95%) in the dementia group showed FLAIR high-intensity signals in the deep subcortical white matter, whereas only 21 of 39 patients (53.85%) showed high-intensity in the white matter with episodic symptoms. There was a significant difference between dementia and episodic symptoms in patients with leukoencephalopathy according to the chi-square test (*p* = 0.0377) (Table 1). The mean size of the GGC repeat sequence was 110.20. There were no significant differences in the GGC repeat sequences and age of onset between the clinical manifestations according to the Kruskal–Wallis test (Figures 3A,B). In addition, our study did not detect a correlation between the age at onset and the number of GGC repeats using simple linear regression (Figure 3C). Previous study reported that dementia could account for the main clinical symptoms in NIID (Cao et al., 2022; Liu et al., 2022; Tai et al., 2023) and some report showed that MRI signal changes could be seen in parallel with patients’ cognitive decline (Abe and Fujita, 2017; Tamura et al., 2021). There was also the report that the brain pathology of NIID has been associated with areas of neurodegeneration and MRI signal changes (Yokoi et al., 2016; Kim et al., 2011). To assess the effect of dementia on DWI high-intensity signals, we divided all 112 cases into dementia-positive and dementia-negative groups because dementia was the most common symptom that could affect MRI signal changes. The number of DWI high-intensity signals-positive cases was significantly higher in the dementia-positive group than in the dementia-negative group (*p* = 0.019) (Supplementary Table S1). Dementia was also frequently observed in the autonomic failure group. We divided the cases having autonomic failure into two groups: dementia-positive and dementia-negative. We found that the rate of patients with DWI high-intensity signal intensity was significantly higher in the dementia-positive group (*p* < 0.0010) (Supplementary Table S2). To adjust for the potential confounders of sex ratio and age of onset, we performed multiple logistic regression analysis with sex ratio, age of onset, and cognitive function as independent variables on 112 cases to examine their association with DWI and T2/FLAIR signal change. The results of the regression analysis showed that the effects of age of onset and sex ratio were not significant for DWI high signal (Sex ratio; Odds ratio = 1.204, *p* = 0.725. Age of onset; Odds ratio = 1.025, *p* = 0.1896. Dementia; Odds ratio = 4.619, *p* = 0.0047) (Supplementary Table S3), and that cognitive impairment was significantly associated with DWI signal change. On the other hand, both sex ratio and cognitive function were associated with T2/FLAIR signal change, suggesting that sex ratio may be a confounding factor (Sex ratio; Odds ratio = 3.207, *p* = 0.0181. Age of onset; Odds ratio = 1.029, *p* = 0.1132. Dementia; Odds ratio = 7.217, *p* < 0.0001) (Supplementary Table S3). Even after adjustment for sex ratio and age of onset, the odds ratio suggested that cognitive function had the strongest effect on both (Supplementary Table S3).

## 5 Discussion

A notable aspect of our study is that we analyzed MRI changes in a patient with NIID over 10 years and explained the correlation between MRI characteristics and clinical manifestations in our case and the literature. We discovered that DWI high-intensity signal expansion in the CMJ is related to cognitive decline and that DWI high-intensity signals in the CMJ are not essential for patients without dementia. In cases with cognitive decline, the rate of patients with FLAIR high-intensity signals in the deep subcortical white matter was also high. When both DWI and FLAIR show high-intensity signals, neurons exhibit spongiotic changes. In contrast, when only the FLAIR image intensity signal was high, slight damage to the myelin sheath was observed (Yokoi et al., 2016). The present case showed FLAIR hyperintensity signals in the white matter at an early stage and did not show severe cognitive decline. However, after the expansion of DWI high-intensity signals, the patient showed progressive cognitive decline, suggesting that severe neuron loss had occurred alongside the brain DWI high-intensity signals expansion. Diffuse high signals in the CMJ on DWI are produced as the disease advances due to irreversible spongiform degeneration (Kim et al., 2011), although the exact mechanism underlying DWI high-intensity signals has not yet been elucidated. Mechanistic hypotheses include neuronal degeneration, T2 shine-through, brain edema, and brain hyperperfusion. A subcortical lesion with FLAIR high-intensity signals without DWI high-intensity signals could result from mild myelin loss, which would not cause severe cognitive decline. Pathological findings of subcortical lesions exhibiting astrocyte loss (Yokoi et al., 2016) and vasogenic edema or hyperperfusion of the subcortex could cause FLAIR high-intensity signal lesions in the white matter (Tai et al., 2022). We suggest that our patient initially had mild myelin or astrocyte loss in the deep white matter and mild neuron loss in the cortical lesion in 2014, and severe neuronal loss gradually progressed in association with expanded high-intensity signals on DWI.

In our literature review, we found some correlations between clinical manifestations and MRI findings. Dementia constitutes the most common symptom in NIID cases (Tai et al., 2023). High-intensity DWI signals and leukoencephalopathy were observed in most patients with dementia. Notably, the number of dementia patients with high-intensity DWI signals was significantly higher than that of patients without dementia (Supplementary Table S1). In other clinical symptom groups, high-intensity DWI signals in the CMJ were observed; however, no statistical differences were detected (Table 1). The reason why high-intensity DWI signals were detected in all types is that NIID is a multisystem disorder, and eosinophilic intranuclear inclusion complexes were detected in the central nervous system, peripheral nervous system, and other visceral organs. The movement disorder, autonomic failure and episodic symptoms groups include cognitive impairment at 8.70, 82.76 and 53.85%, respectively (Table 1). Our data suggest that cognitive impairment in each case affected the frequency of high-intensity DWI signals.

Leukoencephalopathy was also detected in the movement disorder and autonomic failure groups. Only the episodic symptom group showed statistical differences relative to dementia type (Table 1). The number of patients having episodic symptoms with leukoencephalopathy is lower than that with dementia.

Previous studies described that some NIID cases did not show high-intensity DWI signals, even though the GGC repeat of *NOTHCH2NLC* expanded (Wang et al., 2022; Lee et al., 2024). Some possible explanations were suggested for DWI negativity (Liu et al., 2023). DWI high-intensity signals are not observed throughout the follow-up period, or they are initially negative before turning positive (Liu et al., 2023). Spongy degeneration or neuronal loss may not occur during the follow-up period. According to previous research, the majority of neurons in all neocortical areas with eosinophilic intranuclear inclusions had not displayed spongy degeneration (McFadden et al., 2005). Myelin or astrocytes may be initially damaged, followed by spongy neuronal degeneration after glial cell damage at a late stage. Recently, a few NIID cases have shown normal DWI signals with expansion of the GGC repeat sequence (Kim et al., 2011). High-intensity FLAIR signals with normal-intensity DWI signals may indicate glial cell damage caused by eosinophilic intranuclear inclusion complexes, suggesting initial steps toward neuronal degeneration in the brain. Cognitive function may decline even in dementia-negative populations as they get older, and high-intensity DWI signals may be observed according to cognitive decline due to neuron loss.

This observational study reveals a correlation between clinical manifestation and MRI features of NIID. Patients with NIID are divided into four subgroups using hierarchical clustering. According to our 10-year follow-up study and literature review, we found that some clinical features are closely associated with MRI features. While our study includes only 112 cases and NIID is a rare disease, some data including relationship between MRI feature and clinical subgroups could not show significant differences and this may be due to the limited sample size. We thought more cases need to be investigated to explore the relationship between clinical manifestations and MRI features in more detail.

A high-intensity DWI signal is a strong indicator of NIID; however, DWI signals may depend on the timing of imaging or clinical manifestations. Some patients diagnosed with Parkinson’s disease or peripheral neuropathy may never be considered as having NIID and may not undergo an additional MRI examination. Given that our patient showed DWI signal changes, a follow-up brain MRI should be considered in cases of neurological disease to diagnose NIID more frequently.

## Data Availability

The original contributions presented in the study are included in the article/Supplementary material, further inquiries can be directed to the corresponding author.

## References

[ref1] Abe K. Fujita M. (2017). Over 10 years MRI observation of a patient with neuronal intranuclear inclusion disease. BMJ Case Rep. 2017:bcr2016218790. doi: 10.1136/bcr-2016-218790, PMID: 28237949 PMC5337643

[ref2] Cao Y. Wu J. Yue Y. Zhang C. Liu S. Zhong P.. (2022). Expanding the clinical spectrum of adult-onset neuronal intranuclear inclusion disease. Acta Neurol. Belg. 122, 647–658. doi: 10.1007/s13760-021-01622-4, PMID: 33625684

[ref3] Deng J. Gu M. Miao Y. Yao S. Zhu M. Fang P.. (2019). Long-read sequencing identified repeat expansions in the 5′UTR of the *NOTCH2NLC* gene from Chinese patients with neuronal intranuclear inclusion disease. J. Med. Genet. 56, 758–764. doi: 10.1136/jmedgenet-2019-106268, PMID: 31413119

[ref4] Deng W. P. Yang Z. Huang X. J. Jiang J. W. Luan X. H. Cao L. (2021). Case report: Neuronal intranuclear inclusion disease with oromandibular dystonia onset. Front. Neurol. 12:618595. doi: 10.3389/fneur.2021.618595, PMID: 33679585 PMC7928273

[ref5] Guo J. J. Wang Z. Y. Wang M. Jiang Z. Z. Yu X. F. (2020). Neuronal intranuclear inclusion disease mimicking acute cerebellitis: a case report. World J. Clin. Cases 8, 6122–6129. doi: 10.12998/wjcc.v8.i23.6122, PMID: 33344613 PMC7723690

[ref6] Huang Y. Jin G. Zhan Q. L. Tian Y. Shen L. (2021). Adult-onset neuronal intranuclear inclusion disease, with both stroke-like onset and encephalitic attacks: a case report. BMC Neurol. 21:142. doi: 10.1186/s12883-021-02164-1, PMID: 33789591 PMC8011180

[ref7] Ishihara T. Okamoto T. Saida K. Saitoh Y. Oda S. Sano T.. (2020). Neuronal intranuclear inclusion disease presenting with an MELAS-like episode in chronic polyneuropathy. Neurol. Genet. 6:e531. doi: 10.1212/NXG.0000000000000531, PMID: 33324757 PMC7713717

[ref8] Ishiura H. Shibata S. Yoshimura J. Suzuki Y. Qu W. Doi K.. (2019). Noncoding CGG repeat expansions in neuronal intranuclear inclusion disease, oculopharyngodistal myopathy and an overlapping disease. Nat. Genet. 51, 1222–1232. doi: 10.1038/s41588-019-0458-z, PMID: 31332380

[ref9] Ishizawa K. Komori T. Homma T. Sone J. Nakata Y. Nakazato Y.. (2024). The predominance of “astrocytic” intranuclear inclusions in neuronal intranuclear inclusion disease manifesting encephalopathy-like symptoms: a case series with brain biopsy. Neuropathology 44, 351–365. doi: 10.1111/neup.12971, PMID: 38477063

[ref10] Kim J. H. Choi B. S. Jung C. Chang Y. Kim S. (2011). Diffusion-weighted imaging and magnetic resonance spectroscopy of sporadic Creutzfeldt–Jakob disease: correlation with clinical course. Neuroradiology 53, 939–945. doi: 10.1007/s00234-010-0820-4, PMID: 21221559

[ref11] Lee G. H. Jung E. Jung N. Y. Mizuguchi T. Matsumoto N. Kim E. J. (2024). Case report: Neuronal intranuclear inclusion disease initially mimicking reversible cerebral vasoconstriction syndrome: serial neuroimaging findings during an 11-year follow-up. Front. Neurol. 15:1347646. doi: 10.3389/fneur.2024.1347646, PMID: 38405405 PMC10884197

[ref12] Li M. Li K. Li X. Tian Y. Shen L. Wu G.. (2020). Multiple reversible encephalitic attacks: a rare manifestation of neuronal intranuclear inclusion disease. BMC Neurol. 20:125. doi: 10.1186/s12883-020-01712-5, PMID: 32268889 PMC7140360

[ref13] Liang H. Wang B. Li Q. Deng J. Wang L. Wang H.. (2020). Clinical and pathological features in adult-onset NIID patients with cortical enhancement. J. Neurol. 267, 3187–3198. doi: 10.1007/s00415-020-09945-7, PMID: 32535679

[ref14] Liao Y. C. Chang F. P. Huang H. W. Chen T. B. Chou Y. T. Hsu S. L.. (2022). GGC repeat expansion of *NOTCH2NLC* in Taiwanese patients with inherited neuropathies. Neurology 98, e199–e206. doi: 10.1212/WNL.0000000000013008, PMID: 34675106

[ref15] Lindenberg R. Rubinstein L. J. Herman M. M. Haydon G. B. (1968). A light and electron microscopy study of an unusual widespread nuclear inclusion body disease. A possible residuum of an old herpesvirus infection. Acta Neuropathol. 10, 54–73. doi: 10.1007/BF00690510, PMID: 4295804

[ref16] Liu D. Chen K. Tan S. Yin L. L. Li M. Wang Y. S. (2023). Longitudinal course of hyperintensity on diffusion weighted imaging in adult-onset neuronal intranuclear inclusion disease patients. Front. Neurol. 14:1178307. doi: 10.3389/fneur.2023.1178307, PMID: 37404945 PMC10315630

[ref17] Liu Y. H. Chou Y. T. Chang F. P. Lee W. J. Guo Y. C. Chou C. T.. (2022). Neuronal intranuclear inclusion disease in patients with adult-onset non-vascular leukoencephalopathy. Brain 145, 3010–3021. doi: 10.1093/brain/awac135, PMID: 35411397

[ref18] McFadden K. Hamilton R. Insalaco S. Lavine L. Al-Mateen M. Wang G. (2005). Neuronal intranuclear inclusion disease without polyglutamine inclusions in a child. J. Neuropathol. Exp. Neurol. 64, 545–552. doi: 10.1093/jnen/64.6.545, PMID: 15977647 PMC1402362

[ref19] Michaud J. Gilbert J. J. (1981). Multiple system atrophy with neuronal intranuclear hyaline inclusions. Report of a new case with light and electron microscopic studies. Acta Neuropathol. 54, 113–119. doi: 10.1007/BF00689403, PMID: 6264727

[ref20] Miyaue N. Ochi C. Ito Y. H. Ando R. Sone J. Nagai M. (2024). Blepharoptosis as an early manifestation of neuronal intranuclear inclusion disease. Intern. Med. 63, 1163–1166. doi: 10.2169/internalmedicine.2384-23, PMID: 38616117 PMC11081893

[ref21] Okubo M. Doi H. Fukai R. Fujita A. Mitsuhashi S. Hashiguchi S.. (2019). GGC repeat expansion of *NOTCH2NLC* in adult patients with leukoencephalopathy. Ann. Neurol. 86, 962–968. doi: 10.1002/ana.25586, PMID: 31433517

[ref22] Pang J. Yang J. Yuan Y. Gao Y. Shi C. Fan S.. (2021). The value of *NOTCH2NLC* gene detection and skin biopsy in the diagnosis of neuronal intranuclear inclusion disease. Front. Neurol. 12:624321. doi: 10.3389/fneur.2021.624321, PMID: 34017298 PMC8129528

[ref23] Schuffler M. D. Bird T. D. Sumi S. M. Cook A. (1978). A familial neuronal disease presenting as intestinal pseudoobstruction. Gastroenterology 75, 889–898. doi: 10.1016/0016-5085(78)90476-6, PMID: 212342

[ref24] Sone J. Mitsuhashi S. Fujita A. Mizuguchi T. Hamanaka K. Mori K.. (2019). Long-read sequencing identifies GGC repeat expansions in *NOTCH2NLC* associated with neuronal intranuclear inclusion disease. Nat. Genet. 51, 1215–1221. doi: 10.1038/s41588-019-0459-y, PMID: 31332381

[ref25] Sone J. Mori K. Inagaki T. Katsumata R. Takagi S. Yokoi S.. (2016). Clinicopathological features of adult-onset neuronal intranuclear inclusion disease. Brain 139, 3170–3186. doi: 10.1093/brain/aww249, PMID: 27797808 PMC5382941

[ref26] Tai H. F. Hua T. T. Zhang Z. Q. Duan Y. Y. Zhuo Z. Z. Wang A.. (2022). Characteristic cerebral perfusion pattern in neuronal intranuclear inclusion disease. Front. Neurosci. 16:1081383. doi: 10.3389/fnins.2022.1081383, PMID: 36570826 PMC9768440

[ref27] Tai H. Wang A. Zhang Y. Liu S. Pan Y. Li K.. (2023). Clinical features and classification of neuronal intranuclear inclusion disease. Neurol. Genet. 9:e200057. doi: 10.1212/NXG.0000000000200057, PMID: 37090934 PMC10117695

[ref28] Takahashi-Fujigasaki J. (2003). Neuronal intranuclear hyaline inclusion disease. Neuropathology 23, 351–359. doi: 10.1046/j.1440-1789.2003.00524.x 14719553

[ref29] Tamura A. Fujino Y. Sone J. Shiga K. (2021). Temporal changes in brain magnetic resonance imaging findings over 16 years in a patient with neuronal intranuclear inclusion disease. Intern. Med. 60, 2483–2486. doi: 10.2169/internalmedicine.6371-20, PMID: 33642482 PMC8381180

[ref30] Tian Y. Wang J. L. Huang W. Zeng S. Jiao B. Liu Z.. (2019). Expansion of human-specific GGC repeat in neuronal intranuclear inclusion disease-related disorders. Am. J. Hum. Genet. 105, 166–176. doi: 10.1016/j.ajhg.2019.05.013, PMID: 31178126 PMC6612530

[ref31] Wang H. Feng F. Liu J. Deng J. Bai J. Zhang W.. (2022). Sporadic adult-onset neuronal intranuclear inclusion disease without high-intensity signal on DWI and T2WI: a case report. BMC Neurol. 22:150. doi: 10.1186/s12883-022-02673-7, PMID: 35459160 PMC9027041

[ref32] Wang X. J. Qiu X. (2023). Neuronal intranuclear inclusion disease in a 66-year-old woman. Asian J. Surg. 46, 5664–5665. doi: 10.1016/j.asjsur.2023.08.071, PMID: 37625964

[ref33] Xie F. Hu X. Liu P. Zhang D. (2022). A case report of neuronal intranuclear inclusion disease presenting with recurrent migraine-like attacks and cerebral edema: a mimicker of MELAS. Front. Neurol. 13:837844. doi: 10.3389/fneur.2022.837844, PMID: 35299615 PMC8920963

[ref34] Xu L. Zhang H. Yuan H. Xie L. Zhang J. Liang Z. (2023). Not your usual neurodegenerative disease: a case report of neuronal intranuclear inclusion disease with unconventional imaging patterns. Front. Neurosci. 17:1247403. doi: 10.3389/fnins.2023.1247403, PMID: 37638306 PMC10447982

[ref35] Yokoi S. Yasui K. Hasegawa Y. Niwa K. Noguchi Y. Tsuzuki T.. (2016). Pathological background of subcortical hyperintensities on diffusion-weighted images in a case of neuronal intranuclear inclusion disease. Clin. Neuropathol. 35, 375–380. doi: 10.5414/NP300961, PMID: 27719745

[ref36] Yu D. Li J. Tai H. Ma J. Zhang Z. Tang W. (2024). Neuronal intranuclear inclusion disease misdiagnosed as Parkinson’s disease: a case report. J. Int. Med. Res. 52:3000605241233159. doi: 10.1177/03000605241233159, PMID: 38436278 PMC10913512

