# Supplementary material for: Case report: 10-year follow-up of a patient with neuronal intranuclear inclusion disease and a literature review
Source: Front Neurosci. 2025 Jan 15;18:1530160. doi: 10.3389/fnins.2024.1530160 (PMC11774858; doi:10.3389/fnins.2024.1530160)
Supplement: Supplementary file 1 [file Table_1.docx]

Supplementary Material

# Supplementary Table 1

|  | Dementia (+) | | Dementia (-) | |
| --- | --- | --- | --- | --- |
| Sex ratio (ratio of females) | 54.41 | | 59.09 | |
| Age of onset (years; 14 to 86) | 59.10 ± 12.09 (18 to 86) | | 56.36 ± 14.45 (14 to 80) | |
| Total (N = 112) | N = 68 | (%) | N = 44 | (%) |
| **Cognitive impairment** | 68 | 100.00 | 0 | 0.00 |
| Headache | 13 | 19.12 | 13 | 29.55 |
| Dizziness | 7 | 10.29 | 3 | 6.82 |
| Vision disorder | 6 | 8.82 | 6 | 13.64 |
| Ataxia | 12 | 17.65 | 10 | 22.73 |
| **movement disorder** | 29 | 42.65 | 16 | 36.36 |
| Tremor | 28 | 41.18 | 15 | 34.09 |
| Rigidity | 5 | 7.35 | 3 | 6.82 |
| Bradykinesia | 2 | 2.94 | 1 | 2.27 |
| **Autonomic** | 35 | 51.47 | 10 | 22.73 |
| Urinary disturbance | 25 | 36.76 | 4 | 9.09 |
| Constipation | 6 | 8.82 | 0 | 0.00 |
| Syncope | 1 | 1.47 | 2 | 4.55 |
| Miosis | 13 | 19.12 | 3 | 6.82 |
| **Weakness** | 44 | 64.71 | 19 | 43.18 |
| Peripheral neuropathy | 23 | 33.82 | 7 | 15.91 |
| Muscle Weakness | 25 | 36.76 | 12 | 27.27 |
| Sensory disturbance | 12 | 17.65 | 7 | 15.91 |
| **episodic symptom** | 33 | 48.53 | 21 | 47.73 |
| encephalitic episode | 25 | 36.76 | 14 | 31.82 |
| disturbance of Consciousness | 15 | 22.06 | 7 | 15.91 |
| Stroke-like episode | 4 | 5.88 | 4 | 9.09 |
|  | N | score | N | score |
| MMSE (2 to 29) | 43 | 18.74 ± 6.894  (2 to 28) | 16 | 26.13 ± 2.655  (19 to 29) |
| MoCA (9 to 27) | 10 | 16.40 ± 4.835  (9 to 21) | 7 | 22.86 ± 2.854  (20 to 27) |
| FAB (2 to 15) | 14 | 9.36 ± 4.343  (2 to 15) | 2 | 11.50± 4.950  (8 to 15) |
|  | N = 68 | (%) | N = 44 | (%) |
| DWI | 62 | 91.18 | 30 | 68.18 |
| FLAIR | 58 | 85.29 | 21 | 47.73 |
| ventricular distention | 22 | 32.35 | 5 | 11.36 |

**Supplementary Table 1.** Patients were divided into two groups: dementia-positive and dementia-negative. DWI high intensity was significantly increased in dementia by Chi-square (*p* = 0.0019). FLAIR high-intensity was significantly increased in the dementia-positive group, as shown by Chi-square (*p* < 0.0001).

DWI, diffusion-weighted imaging; FAB, frontal assessment battery; MoCA-J, Montreal Cognitive Assessment; MMSE, Mini-Mental State Examination. The mean ± standard error of deviation and range for continuous variables are presented.

# Supplementary Table 2

| Autonomic failure | Dementia (+) | | Dementia (-) | |
| --- | --- | --- | --- | --- |
| Sex ratio (ratio of females) | 45.83 | | 40.00 | |
| Age of onset (years; 27 to 76) | 60.88 ± 10.94 (27 to 76) | | 56.40 ± 7.232 (48 to 67) | |
| Total (N = 29) | N = 24 | (%) | N = 5 | (%) |
| **Cognitive impairment** | 24 | 100.00 | 0 | 0.00 |
| Headache | 4 | 16.67 | 4 | 80.00 |
| Dizziness | 3 | 12.50 | 1 | 20.00 |
| Vision disorder | 1 | 4.17 | 0 | 0.00 |
| Ataxia | 5 | 20.83 | 0 | 0.00 |
| **movement disorder** | 11 | 45.83 | 2 | 40.00 |
| Tremor | 11 | 45.83 | 1 | 20.00 |
| Rigidity | 1 | 4.17 | 0 | 0.00 |
| Bradykinesia | 1 | 4.17 | 1 | 20.00 |
| **Autonomic** | 24 | 100.00 | 5 | 100.00 |
| Urinary disturbance | 17 | 70.83 | 0 | 0.00 |
| Constipation | 4 | 16.67 | 3 | 0.00 |
| Syncope | 1 | 4.17 | 1 | 20.00 |
| Miosis | 9 | 37.50 | 3 | 60.00 |
| **Weakness** | 20 | 83.33 | 3 | 60.00 |
| Peripheral neuropathy | 12 | 50.00 | 2 | 40.00 |
| Muscle Weakness | 11 | 45.83 | 1 | 20.00 |
| Sensory disturbance | 7 | 29.17 | 1 | 20.00 |
| **episodic symptom** | 9 | 37.50 | 1 | 20.00 |
| encephalitic episode | 5 | 20.83 | 0 | 0.00 |
| disturbance of Consciousness | 5 | 20.83 | 1 | 20.00 |
| Stroke-like episode | 0 | 0.00 | 0 | 0.00 |
|  | N | score | N | score |
| MMSE (2 to 28) | 14 | 15.79 ± 6.216  (2 to 25) | 4 | 25.00 ± 4.243  (19 to 28) |
| MoCA (19 to 24) | 1 | 19.00 | 2 | 23.00 ± 1.414  (22 to 24) |
| FAB (2 to 15) | 5 | 8.80 ± 5.541  (2 to 15) | 1 | 8.00 |
|  | N = 24 | (%) | N = 5 | (%) |
| DWI | 23 | 95.83 | 2 | 40.00 |
| FLAIR | 23 | 95.83 | 4 | 80.00 |
| ventricular distention | 12 | 50.00 | 1 | 20.00 |

**Supplementary Table 2.** Patients classified as having autonomic failure were divided into two groups: dementia-positive and dementia-negative. DWI high-intensity was significantly increased in dementia-positive patients, as indicated by Chi-square (*p* = 0.0010). No statistical differences were detected in FLAIR (*p* = 0.2037).

DWI, diffusion-weighted imaging; FAB, frontal assessment battery; MoCA-J, Montreal Cognitive Assessment; MMSE, Mini-Mental State Examination. The mean ± standard error of deviation and range for continuous variables are presented.

# Supplementary Table 3

| Dependent variable: DWI | Odds ratios | 95% CI | P value |
| --- | --- | --- | --- |
| Sex ratio | 1.204 | 0.4215 to 3.412 | 0.7252 |
| Age of onset | 1.025 | 0.9876 to 1.063 | 0.1896 |
| Dementia | 4.619 | 1.658 to 14.28 | 0.0047 |
| Dependent variable: FLAIR | Odds ratios | 95% CI | P value |
| Sex ratio | 3.207 | 1.253 to 8.811 | 0.0181 |
| Age of onset | 1.029 | 0.9936 to 1.068 | 0.1132 |
| Dementia | 7.217 | 2.865 to 19.80 | <0.0001 |

**Supplementary Table 3.** Multiple logistic regression analysis with sex ratio, age of onset, and cognitive function as independent variables on 112 cases were performed to examine their association with DWI and T2/FLAIR signal change. The results of the regression analysis showed that the effects of cognitive impairment was significantly associated with DWI signal change. (Sex ratio; Odds ratio = 1.204, *p* = 0.725. Age of onset; Odds ratio = 1.025, *p* = 0.1896. Dementia; Odds ratio = 4.619, *p* = 0.0047). Both sex ratio and cognitive function were associated with T2/FLAIR signal change (Sex ratio; Odds ratio = 3.207, *p* = 0.0181. Age of onset; Odds ratio = 1.029, *p* = 0.1132. Dementia; Odds ratio = 7.217, *p* < 0.0001).
